# Optimization of ultrasonic pretreatment and analysis of chlorogenic acid in potato leaves

**DOI:** 10.1038/s41598-024-61139-7

**Published:** 2024-05-09

**Authors:** Xin Wang, Xianyun Gong, Binbin Lin

**Affiliations:** Department of Food Engineering, Chemistry, Harbin University, Harbin, Heilongjiang China

**Keywords:** Chlorogenic acid, Potato leaf, Orthogonal experiment, Traditional Chinese medicines, Gauss calculation, Extraction, Chemical biology, Molecular medicine, Chemistry, Materials science

## Abstract

Chlorogenic acid (CA) is an effective ingredient that can strengthen immunity during following the COVID-19 era. The current cost of CA is high owing to its complex purification process and low yield (approximately 2%). In this study, a one-step path orthogonal experiment was designed based on the results from Gauss calculation, which consisted of acidity, coordination, and hydrolysis in molecules. The optimized extraction conditions were 60 ℃, 60 min, 1:20 liquid ratio, and 40% ethanol in a nitrogen atmosphere controlled using a device of our own design, which led to CA yields of up to 6.35% from potato leaves. The purified CA was analyzed using high-performance liquid chromatography, thin-layer chromatography, ultraviolet–visible spectroscopy, and molecular fluorescence. This accurate and reproducible method can not only be used to obtain high yields of CA but can also be used for the quality control of active plant products and their isomers.

## Introduction

Traditional Chinese medicine has taken the stage in the post-epidemic COVID-19 era. Honeysuckle played an extremely important role in the prevention and control of the epidemic. Studies have shown that chlorogenic acid (CA) in honeysuckle can inhibit bacteria and viruses^1–5^. Currently, CA is expensive owing to limited raw material resources and low yields. Thus, there is an urgent to identify substitutes for honeysuckle to ensure a steady yet inexpensive source of CA. Meanwhile, improving the extraction process to increase CA yields could be a suitable approach.

The increased use of honeysuckle in Chinese medicine cannot meet the current demands. Apart from honeysuckle, other traditional Chinese medicines, such as *Eucommia* and *Centella asiatica*, and foods such as potato leaves (PLs) and coffee beans are known to contain a certain amount of CA^6^. The study showed that CA from *Eucommia ulmoides* leaves had an anti-inflammatory effect similar to that of hyaluronidase and stronger than that of aspirin^7^. PLs are used for both medicinal and edible purposes; they are abundant and can be easily obtained by simple planting techniques. The leaves of this perennial trailing herb contain a large amount of CA and numerous effective ingredients such as flavonoids, polysaccharides, and proteins, which can strengthen immunity, delay aging, reduce blood sugar levels, facilitate urination, and prevent night blindness^8,9^.

Methods such as water extraction, organic solvent extraction, fractional extraction, ultrasonic-assisted extraction, microwave-assisted extraction, supercritical CO_2_ extraction, and biological enzyme methods have been evaluated for the extraction of CA derivatives^3,10–14^. Mechanistic studies show the dominant role of CA among the six isomers^15–17^. CA can be purified using traditional, complex extraction methods. As opposed to rotary evaporation and resin separation, the development of a simpler method for the isolation and purification of CA is now gaining considerable attention.

In this study, we provide a method for the extraction of CA (C_16_H_18_O_9_ CAS No. 327-97-9) from PLs using a self-designed device^18^. The extraction conditions were designed based on the results from Gauss calculation. The optimized extraction conditions were verified using orthogonal experiments and high-performance liquid chromatography (HPLC). The structure of CA was determined using spectral methods and chromatography and was found to be consistent with the National Standards of China. Our findings could thus facilitate the subsequent separation and mass production of CA isomers. Currently, the findings are applicable to small-scale extraction at the laboratory level. Additional experimental conditions, such as N_2_ pressure, will be adjusted accordingly based on the results in an industrial setting. This study provides a controllable extraction and purification process to obtain CA isomers.

## Methods

### Materials

Potato leaves (Academy of Agricultural Sciences, Heilongjiang, China); ethanol (Yongda Chemical Reagent Co., Ltd., Tianjin, China); chlorogenic acid (Aladdin Biochemical Technology Co., Ltd., Shanghai, China); acetonitrile (Thermo Fisher Scientific Co., Ltd, Shanghai, China); methanol (Thermo Fisher Scientific Co., Ltd., Shanghai, China).

### Instruments and equipment

HPLC (LC-10AT, Shimadzu, Japan); C18 chromatographic column (LC-C18 150 × 4.6 mm, Shimadzu, Japan); thin-layer chromatography (KH-2300, Shanghai Kezhe, China); UV–vis spectrophotometer (UV-255, Shimadzu Instrument Co., Ltd.). Luminescence spectra were measured using a Perkin–Elmer LS55 Luminescence spectrometer at 25 °C. Infrared spectra were recorded in the range of 400–4000 cm^−1^ on a Perkin–Elmer 1730 FTIR spectrophotometer using KBr pellets.

#### Preparation of PL extract

**Figure 1:**
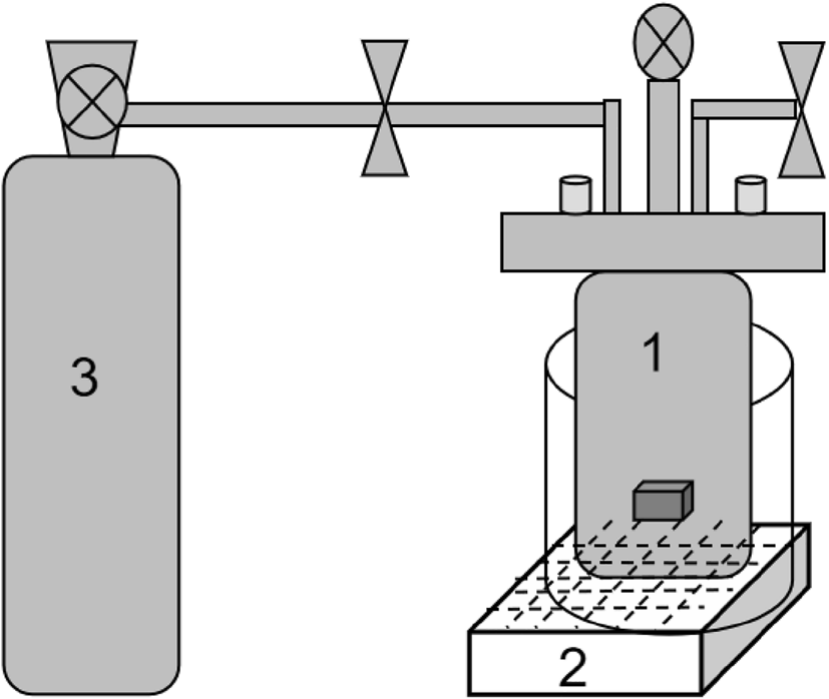
Self-designed device for the extraction of chlorogenic acid in potato leaf powders. Part 1: sealed chamber; Part 2: a device set heat and ultrasonic; Part 3: the compressed gas cylinder of N_2_.

Ground potato leaf powder (0.5 g) was weighed in a round-bottom flask and a mixed solution of absolute ethanol and distilled water was added^19^. This study was focused on finding a set of reaction conditions in a sealed environment of fluid N_2_ that could improve the yield of the active component. As shown in Fig. 1, the samples were prepared in a sealed chamber (part 1). Heating and ultrasonic treatment affected the reaction as N_2_ (part 3) flowed into the chamber. The conditions of the thermal treatment of the mixed solution were investigated by changing the reaction temperature, time, and ratio. To simplify and reduce the number of variables, the following four remarkable factors were proposed: material-to-liquid ratio (1:20, 1:30, 1:40; the solvent was 55% ethanol), extraction time (0.5 h, 1.0 h, 1.5 h), extraction temperature (50 °C, 60 °C, 70 °C), and ethanol volume fraction (40%, 50%, 60%).

#### Preparation of the standard solution

CA (2.0 mg) was dissolved in 50% methanol in a 50 mL volumetric flask to yield a standard solution (40 μg/mL)^19^. Next, different volumes of the standard solution (1 mL, 3 mL, 5 mL, 7 mL, 9 mL) were diluted to 10 mL to obtain a series of concentrations (4 μg/mL, 12 μg/mL, 20 μg/mL, 28 μg/mL, and 36 μg/mL) of the CA standard solution.

#### Purification

The crude extract was purified using AB-8 macroporous adsorption resin as a carrier. The extract was washed and precipitated twice using ethyl acetate and dried in vacuum to obtain pure CA.

#### Chromatographic conditions for HPLC

LC-C18 column (250 mm × 4.6 mm); mobile phase: 0.2% phosphoric acid:acetonitrile (85:15, V/V); detection wavelength: 324 nm; column temperature: 30 °C; injection volume: 20 μL; flow rate: 1.000 mL/min.

#### Gauss calculation

Molecular structure analysis of CA was controlled using Gauss calculation, which consisted of acidity, coordination, harmonic vibrational spectra, natural bond orbital (NBO) analysis and hydrolysis in molecules. The calculation method used the WB97XD/6-31++G* basis sets included in the Gauss03 package. The orbitals were labeled by principal quantum number. A valence basis set consisted of all valence atomic orbitals, occupied or unoccupied, up to the shell of the principal quantum number of the highest occupied valence electrons.

## Results and discussion

### Optimization of extraction factors using orthogonal experiments

**Figure 2:**
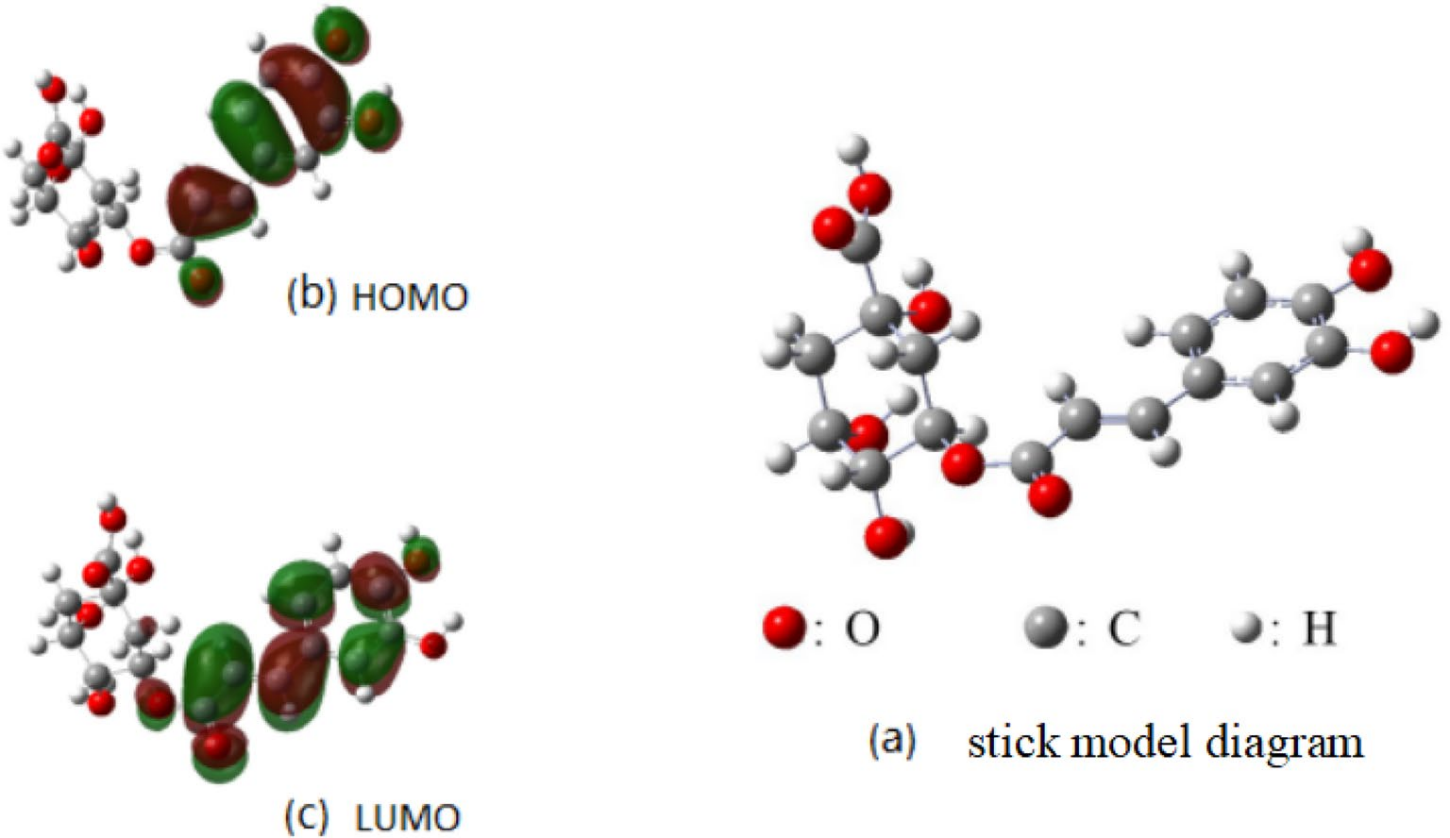
Molecular theoretical calculation diagram of chlorogenic acid: (**a**) stick model diagram; (**b**) HOMO; (**c**) LUMO.

CA was formed by the generation of an ester bond between o-diphenol acrylic acid and polyhydroxy saturated hexacyclic carboxylic acid. The molecule stick model diagram is shown in Fig. 2a. Chemically unstable functional groups such as unsaturated bonds, carboxyl groups, phenolic hydroxyl groups, and ester bonds are present in the structure of CA. Calculations using the frontier orbital theory showed a polyhydroxy saturated six-member ring in the molecular structure presenting as a stable chair configuration and showing distortion at a certain angle between its plane and the phenol acrylate conjugated system. The natural bond orbital diagram has been shown in Fig. 2b, c, presented the usual pattern of frontier molecular orbital in complex. These results included the highest occupied (HOMO) and the lowest unoccupied (LUMO) molecular orbitals for each cluster, which could show the coordination and stability of molecules^20^. The molecule had C1 symmetry. The contributions of the molecular frontier orbitals were attributable to the phenol acrylate conjugated system, and the chemical activity was mainly reflected in the hydroxyl phenol ring and acrylate group. The calculated results indicated its acidity and coordination properties. The hydrogen in the phenol hydroxyl moiety could easily be lost, indicating acidity, whereas the carboxyl oxygen accepted metal cations and showed good coordination ability. Cleavage of the ester bond was helpful in promoting the separation of the saturated six-membered ring owing to its hydrolytic property. Theoretical calculations revealed that CA could decompose, migrate, and rearrange at high temperatures easily. Therefore, temperature and other conditions should be strictly controlled during extraction procedures.

CA and its isomers are widely distributed in many plants. Owing to its pharmacological activity, CA is widely used in the medicine, hygiene, and food and chemical industries. China has abundant plant resources that are rich in CA and its isomers. However, the complex purification processes and low yields need to be addressed. The molecular properties of CA were determined and predicted using the frontier orbital theory of quantum chemistry, and the purified products were qualitatively analyzed using HPLC, thin-layer chromatography, UV–visible spectrophotometry, and molecular fluorescence spectrophotometry.

Based on the results from Gauss calculations, a group of L^9^(3^4^) orthogonal experiments were designed for extraction conditions (Table 1) using the following conditions: extraction temperature: 50, 60, and 70 °C; extraction time: 30, 60, and 90 min; material-to-liquid ratio: 1:20, 1:30, and 1:40; ethanol concentration: 40%, 50%, and 60%. The purified preparation was quantitatively analyzed using HPLC^21^. Based on the variance calculation results from orthogonal experiments, the optimal extraction conditions to obtain CA were determined to be 60 °C for 60 min, 1:20 material-to-liquid ratio, and ethanol concentration of 40%, which increased the yield of CA to 6.35%.

**Table 1:** Orthogonal experiment of ethanol ultrasonic extraction and chlorogenic acid content.

	Extraction temperature/℃	Extraction time/min	Material liquid ratio/g∙mL^−1^	Ethanol concentration/%	Chlorogenic acid content/mg∙g^−1^
1	50	30	1: 20	40	0.1500
2	60	30	1: 30	50	0.1000
3	70	30	1: 40	60	0.1750
4	50	60	1: 40	60	0.1750
5	60	60	1: 20	40	3.1750
6	70	60	1: 30	50	0.0500
7	50	90	1: 30	50	0.0750
8	60	90	1: 40	60	0.4250
9	70	90	1: 20	40	0.6500

### Spectroscopic analysis of CA are consistent with the fitting results

**Figure 3:**
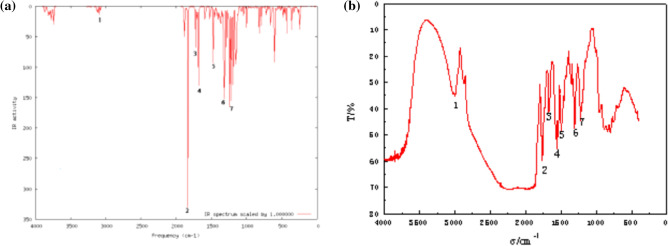
IR spectrum of chlorogenic acid: (**a**) fitting of Gauss03; (**b**) extraction.

Figure 3a shows the infrared spectrum of CA fitted by the harmonic vibrational spectra from Gauss03, and Fig. 3b shows the extraction scheme^20^. Peak 1 indicates the C–H stretching vibration on the benzene ring, whereas peak 2 corresponds to the stretching vibration of the carbonyl group. Absorption peak 3 is indicative of the stretching vibration in C=C. Absorption peaks 4 and 5 represent the vibration of the benzene ring skeleton. Absorption peak 6 is the characteristic absorption of CH_2_ shear bending. The strengthened absorption peak 7 is attributable to the C–O bond (ester, alcohol). These findings were consistent with the fitting results, indicating that the extracted compound was indeed the target compound.

**Figure 4:**
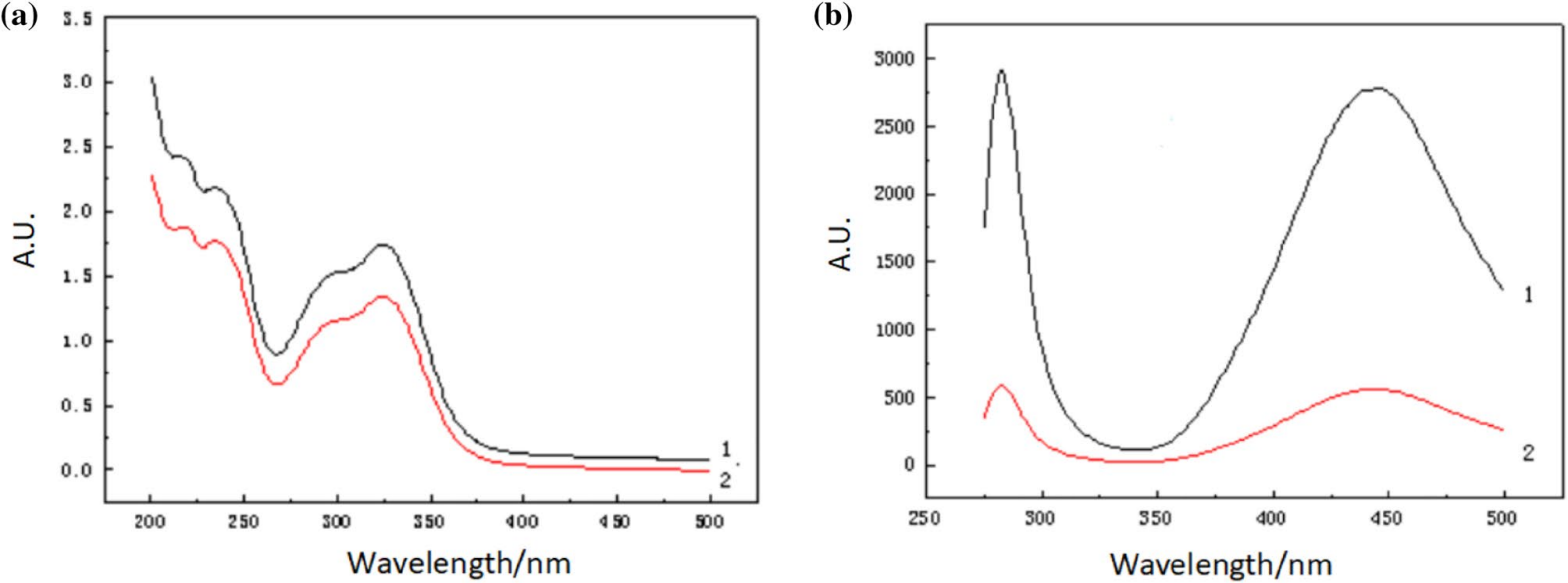
UV spectrum (**a**) and fluorescence spectrum (**b**) of chlorogenic acid: curve 1 is chlorogenic acid standard; curve 2 is extraction.

The diluted PL extract was compared with CA standard solution using 50% methanol as the blank and scanning at a wavelength of 200–500 nm. The maximum absorption was 324 nm, which was consistent with CA standard (Fig. 4a). Figure 4b shows the fluorescence spectrum. The excitation and emission wavelengths of the extract were the same as those of the CA standard at both 279 nm and 445 nm. The findings from UV and fluorescence spectrophotometry confirmed that the extract was indeed CA.

### Determination of CA using chromatography

#### Qualitative analysis by thin-layer chromatography with the optimal developing agent

**Figure 5:**
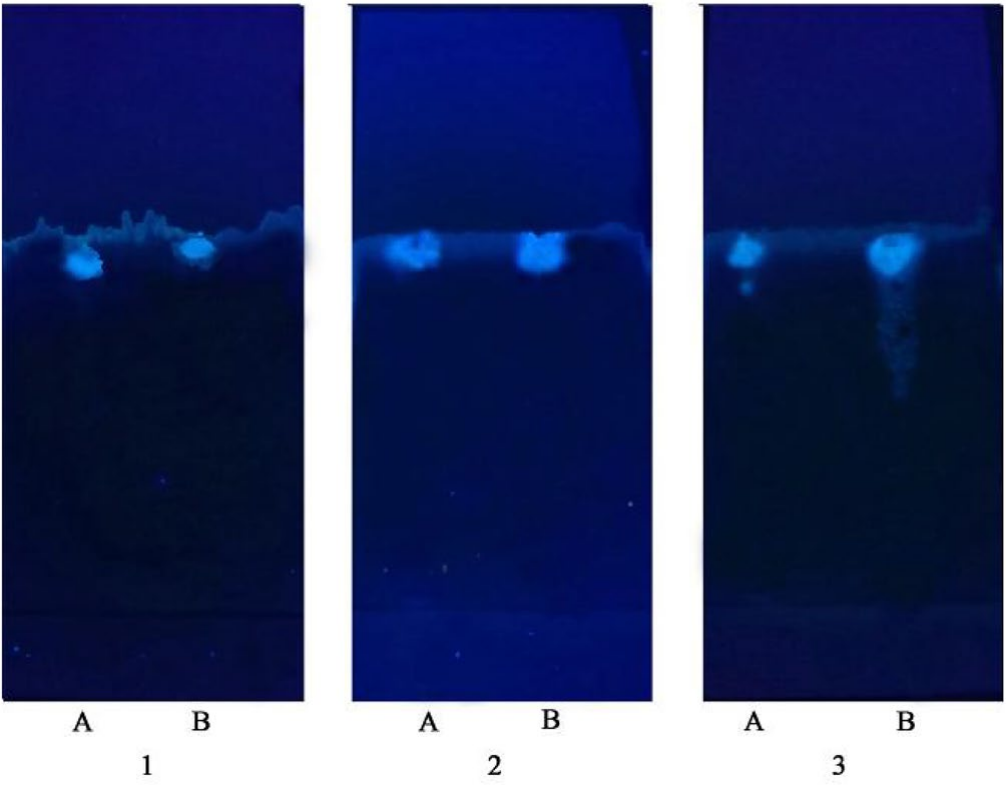
TLC (A: chlorogenic acid standard; B: potato leaf extract) 1: ethyl acetate acetone formic acid water (7:3:2:1, V/V); 2: ethyl acetate acetone formic acid (7:3:2, V/V); 3: ethyl acetate formic acid water (10:3:1, V/V).

The extract and standard samples were spotted on a silica gel GF_254_ thin-layer plate and placed in a tank containing the solvent mixture for development (ethyl acetate:acetone:formic acid:water = 7:3:2:1, ethyl acetate:acetone:formic acid = 7:3:2, and ethyl acetate:formic acid:water = 10:3:1, V/V), dried, and observed at 254 nm in an ultraviolet chamber. The spots of CA (Fig. 5a) are clearer but are not on the same horizontal line as those of the extract. The spot of CA and the extract appear clearly on the same horizontal line and there is no obvious tailing (Fig. 5b). Although the two spots are on the same horizontal line, they appear slightly blurred and show trailing (Fig. 5c). These findings indicate the lack of abnormal phenomena when observed at 254 nm using an ultraviolet light source. Qualitative analysis using thin-layer chromatography indicated ethyl acetate:acetone:formic acid = 7:3:2 (V/V) as the optimal developing agent.

#### Quantitative analysis by HPLC and well linearity

**Figure 6:**
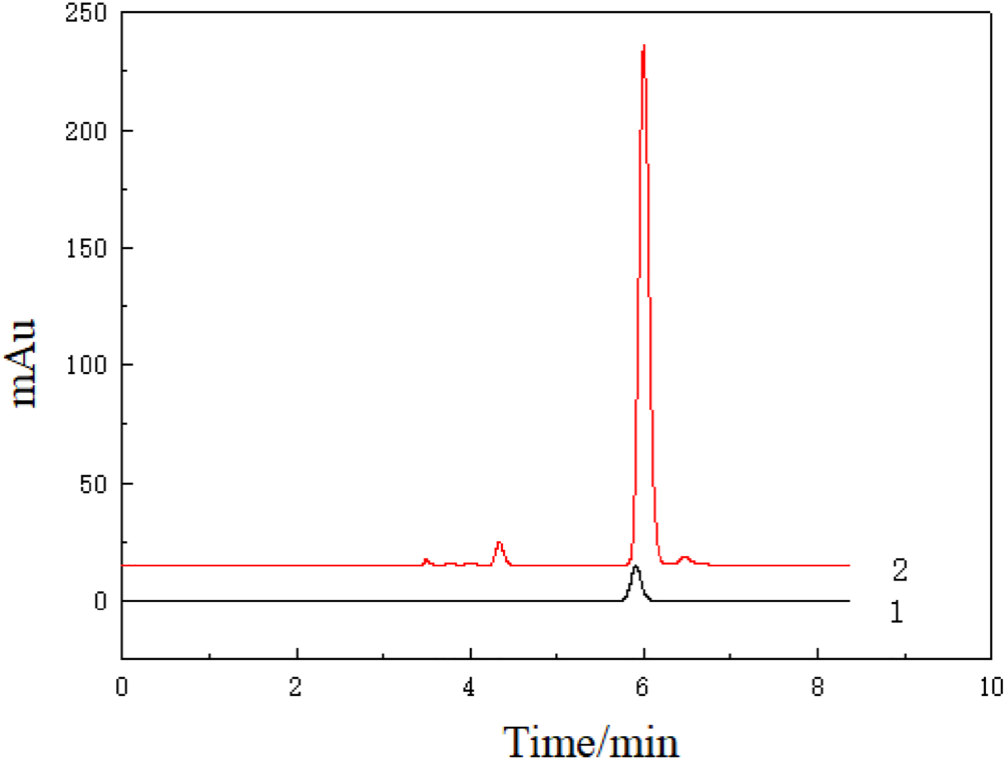
HPLC of chlorogenic acid 1: chlorogenic acid standard; 2: potato leaf extraction.

Figure 6 shows that the retention time of standard CA is the same as that of the extract diluted 250 times, indicating the same chemical composition^22^. The linear regression equation of CA was y = 32452.85x + 10255.98, correlation coefficient R = 0.9927. The results showed that the concentration of CA in the extract was 4–36 μg/mL. A good linear relationship was found, indicating the suitability of the method for quantitative analysis.

## Conclusions

In this paper, a simple pathway to obtain CA has been reported using a self-designed equipment. The extraction temperature should be strictly controlled during the extraction of PLs. An orthogonal experiment was designed to optimize the ultrasonic pretreatment process. The optimal conditions were as follows: extraction temperature of 60 °C, extraction time of 60 min, material-to-liquid ratio of 1:20, and ethanol concentration of 40% in an atmosphere of N_2_. These conditions could increase CA yields by up to 6.35%. Based on the results from theoretical prediction and by optimizing the experimental process, this combination could be effectively used to increase CA yields. Therefore, this simple, reproducible, and highly accurate method can be used to obtain high yields of CA and serves as a reliable experimental basis for the quality control of active plant products and their isomers. Traditional Chinese medicine has a relatively complex composition. CA extracted from PLs was easily purified. However, it is difficult to obtain other isomers of CA such as neochlorogenic acid and cryptochlorogenic acid during the extraction process. Accordingly, future studies can focus on improving the extraction process, optimizing experimental conditions, and establishing controllable extraction procedures to obtain CA isomers. Follow-up studies would involve the separation and purification of CA from other raw materials used as traditional Chinese medicine including *Eucommia ulmoides* and *Centella asiatica* leaves. Further elucidation of the dynamic process and establishment of a model are currently underway.

## Data Availability

All data generated or analysed during this study are included in this published article.
